# Effect of Sleep Intervention Programs during Cardiac Rehabilitation on the Sleep Quality of Heart Patients

**DOI:** 10.1155/2022/8269799

**Published:** 2022-03-24

**Authors:** Fatemeh Ghane, Mahdieh Ghanbari Firoozabadi, Farzan Madadizadeh, Khadijeh Nasiriani

**Affiliations:** ^1^Critical Care Nursing, Department of Nursing, Shahid Sadoughi University of Medical Sciences Campus, Yazd, Iran; ^2^Yazd Cardiovascular Research Center, Shahid Sadoughi University of Medical Sciences, Yazd, Iran; ^3^Center for Healthcare Data Modeling, Departments of Biostatistics and Epidemiology, School of Public Health, Shahid Sadoughi University of Medical Sciences, Yazd, Iran; ^4^Department of Nursing, Research Centre for Nursing and Midwifery Care, Mother and Newbern Health Research Centre, Shahid Sadoughi University of Medical Sciences, Yazd, Iran

## Abstract

**Materials and Methods:**

In this quasi-experimental study with unequal control group design, 35 individuals participated in the cardiac rehabilitation program as the experimental group and 35 served as the control group. The program included 12 weeks of exercise, 3 sessions per week, 3 sessions of training programs each lasting for 45 minutes, and a special two-session sleep improvement program. Data were collected using the Pittsburgh Sleep Quality Index and analysed with descriptive and inferential statistical methods.

**Results:**

There were not any significant differences between the two groups in age, sex, marital status, smoking, and indication for cardiac rehabilitation (*P* > 0.05). The scores of sleep quality of patients were 9.2 ± 1.58 before and 4.40 ± 1.14 after intervention in the experimental group and 9.02 ± 2.56 before and 7.48 ± 1.86 after intervention in the control group. There was no significant difference between the two groups before intervention (*P* = 0.73). yet there was a significant difference after intervention (*P* = 0.0001). In addition, scores of sleep quality of patients were significantly different in the experimental and control groups before and after intervention (*P* = 0.0001).

**Conclusion:**

Findings indicated that the quality of sleep of cardiac patients improved after the sleep intervention program during the cardiac rehabilitation program. Therefore, it is suggested to implement sleep improvement programs for cardiac patient care as an effective, easy, and feasible technique. In addition, it is necessary to pay more attention to the sleep improvement program in cardiac rehabilitation. *Trial Registration*. The trial was retrospectively registered on https://en.irct.ir/trial/50799 on 14 September 2020 (14.09.2020) with registration number IRCT20140307016870N6.

## 1. Introduction

Cardiovascular disease is the most common cause of death and disability in the world and in Iran. Some studies show that patients suffer from sleep disorders (SD) after heart accidents and coronary artery surgery [1]. Sleep is an important modulator of heart function and can thus lead to lower activity and cardiac load, so that SDs such as insomnia and hypersomnia have also been considered important factors in the pathogenesis and progression of cardiovascular diseases [2].

Sleep disorder over a significant period of time can also disrupt many aspects of quality of life, including general health, physical, cognitive, and psychological functions, and daily activities [1, 3]. SD has a prevalence of about 30% in human societies [4]. However, sleep is an important recurring and regular biological period accompanied by physical and mental power regeneration [5]. One-third of human life is spent in sleeping [6]. Sleep physiology is not yet fully understood, but it is widely accepted that sleep is important for the human body in restoring metabolic and neural processes [7]. Sleep and rest are among the physiological and basic needs of human beings, the deprivation of which endangers human life and health leading to conditions such as cardiovascular disease [8]. When sleep is completely or partially limited, important brain functions are affected leading to mental and neurological disorders [7]. Sleep quality (SQ) is defined as one's satisfaction of the sleep experience, integrating aspects of sleep initiation, sleep maintenance, sleep quantity, and refreshment upon awakening [9]. Lower SQ is a common problem in cardiac patients [4]. Chronic diseases account for about half of the global burden of disease, and cardiovascular diseases account for the largest rate [1]. Researchers have tried to identify effective interventions to improve the quality of sleep in these patients. Cardiac rehabilitation (CR) program is another factor that has been suggested for improving quality of sleep in cardiac patients [10].

The sleep intervention program is part of the cardiac rehabilitation program. Cardiac rehabilitation (CR) is defined as a medical care program designed to improve patients' cardiovascular health and function [1]; it is a comprehensive long-term program under the supervision of medical services including medical evaluation, exercise program, modification of cardiac risk factors, training, and counseling [11]. CR has been known as an important measure for the effectiveness of heart disease and reducing its complications and a useful solution to improve quality of life and reduce disability and mortality in cardiovascular diseases [12]. CR can reduce the effects of SD and can improve the sleep cycle of patients by affecting depression and anxiety caused by heart disease. Benefits of exercise programs have been controversial on sleep habits, and they have had positive effects in some cases, while other programs have not had any significant effect [7]. On the other hand, current interventions to treat sleep problems are often inaccessible, costly, and time-consuming [13]; hence, designing a short sleep behavioral intervention program may have significant benefits in improving sleep [14]. In particular, there are few studies on interventions to improve sleep in patients undergoing CR [7]; besides, a majority of studies are conducted on patients admitted to the intensive care unit, and there are few studies on discharged patients. Therefore, it seems necessary to add special sleep improvement programs to the heart rehabilitation program. Moreover, ensuring adequate sleep for patients is a basic task of nurses who should use appropriate nursing measures to manage SD in addition to assessing and determining their causes [15]. Therefore, the present study was conducted to determine the effect of a special sleep intervention program during CR on the quality of sleep in heart patients.

## 2. Methodology

### 2.1. Study Design

This quasi-experimental study used the unequal control group design.

### 2.2. Setting

This study was conducted in Afshar Heart Rehabilitation Center of Yazd, Iran, and compared control and experimental groups.

### 2.3. Sample Size

The sample size was based on a study by Lin et al.

(1) $$\begin{array}{c} n\geq \frac{2\left(z_{1-\left(\alpha /2\right)}+z_{1-\beta }\right)s^{2}}{{\left({\bar{x}}_{1}-{\bar{x}}_{2}\right)}^{2}}=\frac{2{\left(2.57+1.64\right)}^{2}\;28.43}{{\left(45.33-39.27\right)}^{2}}=27. \end{array}$$

Considering type 1 error equal to 0.05 and power of test equal to 0.95, it was obtained equal to 35, considering 25% nonresponse probability [16].

### 2.4. Participants

The research samples were selected from patients with heart problems who were discharged from Afshar Hospital in Yazd, Iran, and referred to the rehabilitation center using a purposive sampling method based on inclusion and exclusion criteria. Then, the subjects were randomly assigned to the intervention and control groups based on the possibility of participating in the cardiac rehabilitation program.

Inclusion criteria were cardiovascular diseases requiring CR, age of 18 years and older, familiarity with the Persian language, and consent to participate in the study.

Exclusion criteria were sleep disorder requiring medication before the rehabilitation, history of hypnotic drug use for more than 6 months, history of depression and anxiety according to a psychiatrist, cognitive impairment (vision or hearing problem), lack of participation in more than one session, loss of consciousness during the intervention, patient's exacerbated clinical condition during CR, and canceling the continuation of the study.

### 2.5. Intervention

**Figure 1:**
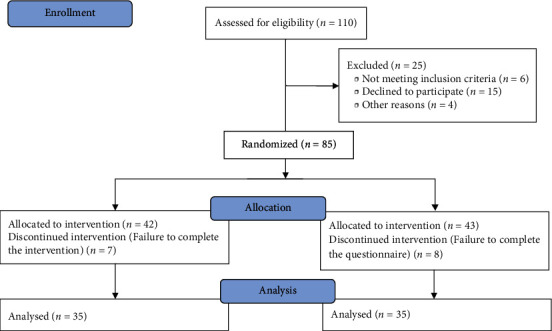
CONSORT flow diagram.

Patients who participated in the rehabilitation program were considered the experimental group, and patients who did not participate in the rehabilitation program or left the program despite being taught the need for rehabilitation were assigned to the control group. In both groups, the selection of samples continued until a sample size of 35 was achieved (Figure 1). After selecting patients and obtaining informed consent to participate in the study, their demographic characteristics and SQ were completed using the Pittsburgh Sleep Quality Index (PSQI). Based on a complete examination and results of the initial exercise test of patients, having a companion, and other factors such as a history of heart surgery, myocardial infarction, and patients' risk levels, the clinician specified the length and speed of exercise with treadmill and exercise program for any patient in the experimental group to perform the rehabilitation program. Accordingly, the intensity of training in the initial sessions started from 20% to 40% of the reserve heart rate and gradually increased to 60% in the last sessions. The CR program included 12 weeks of exercise, 3 sessions per week, and 3 sessions of training programs each for 45 minutes. Three collective question and answer sessions and lecture training were held by a team including a physician, nutritionist, psychologist, and health educator with an emphasis on the role and importance of CR in patients' recovery after cardiac surgery, strategies for correcting cardiac risk factors, diet modification, and adopting a healthy lifestyle including quitting smoking and exercise, improving quality of life, adapting to pain, anxiety, depression, postoperative problems, and sexual activity of patients after a heart attack; then, educational pamphlets were provided for patients. The patients' spouses or families were also invited to participate in training sessions to encourage home exercises and provide social support.

In addition, a special sleep improvement program was implemented in two 45-minute sessions for patients. This program was performed in the third week of rehabilitation by a nurse trained in promoting rest and sleep in sleep-deprived patients. It provided explanation of the following: (1) definition of sleep and importance of adequate sleep, (2) different types of SD, (3) determination of sleep characteristics and habits in the first session, (4) identification of a wrong behavioral pattern relating to sleep, and (5) useful methods of controlling SD, especially sleep improvement methods in heart patients in the second session. Furthermore, videos and photos were displayed, and their experiences were discussed.

### 2.6. Comparisons

Before the intervention, the questionnaire was completed in the first session of cardiac rehabilitation, and of course, according to the patient diagnosis, it was 4 weeks after heart surgery or a week after percutaneous coronary intervention. Due to the fact that the rehabilitation program usually lasts for 3 months, the questionnaires were completed face-to-face for the experimental group and by phone for the control group after 3 months by researchers FG and MGF.

### 2.7. Instruments

The data collection tools included a demographic and clinical profile form (age, sex, marital status, smoking, and indication for CR) and the Pittsburgh Sleep Quality Index (PSQI) [17]. The PSQI examines the quality of sleep over the past month. It has 9 items. Item 5 has 10 subcategories that provide a general description of SQ, sleep delay, useful sleep duration, the ratio of useful sleep duration to the total time spent in bed, sleep disorder, waking up due to the shortness of breath, nocturnal cough, body aches, extreme cold, extreme heat, use of sleeping pills to fall asleep, drowsiness, and inability and nonmotivation to exercise during the day caused by insomnia. All 19 items were given three types of scores to score the PSQI. Note that getting a total score above 5 in the whole PSQI means poor SQ [18]. The PSQI is a standard PSQI for assessing SQ over the past month. The validity and reliability of the PSQI have been proven in several studies. Buysse et al. reported the internal validity of *α* = 0.83 and reliability in the retest to be *r* = 0.85 [17]. Parker et al. also reported a sensitivity of 90% and specificity of 87% [19]; Bertolazi et al. reported the reliability coefficient of *r* = 0.82 with high validity [20]. Furthermore, the reliability of the PSQI was obtained equal to *r* = 0.88 by Hosseinabadi et al. in Iran [21]. The PSQI is available at the following web address: https://www.psychiatry.pitt.edu/sites/default/files/inline-files/PSQI%20Article.pdf [17].

### 2.8. Statistical Analysis

Data analysis was performed using SPSS16. The mean and standard deviation were used to describe quantitative variables of the study in two groups according to the normality of error in variables. The frequency and percentage were reported for qualitative variables. The Kolmogorov–Smirnov test was used to determine the normality of the data distribution. The independent *t*-test and paired *t*-test were used to perform the relevant inferential statistics for comparing the mean scores of SQ in both groups and according to the normality of the SQ score in each group (*P* = 0.05).

### 2.9. Ethics, Endpoint, and Trial Registration

The present study was approved with the code of ethics IR.SSU.REC.1398.200 on 14 January 2020 by the Ethics Committee of Shahid Sadoughi University of Medical Sciences in Yazd. Written consent was obtained from participants in the interventional group. In addition, oral consent was obtained from the participants in the control group. This study was registered at ClinicalTrials.gov (IRCT20140307016870N6) on 14 September 2020.

## 3. Results

A total of 70 people completed the study in the experimental and control groups. According to the results, the mean ages of the experimental group (61.88 ± 7.43 years) and control group (61.71 ± 9.54 years) were not significantly different between the two groups (*P* = 0.93). Furthermore, both groups were not significantly different in terms of gender, marital status, education level, employment status, smoking, and indication for CR (*P* > 0.05); the groups were homogeneous, and it was possible to evaluate the intervention effectiveness (Table 1).

Other findings indicated that mean scores of SQ of the participants in the experimental and control groups did not differ significantly before the study (*P* = 0.73), but mean scores of SQ of the participants were significantly different after the study (*P* = 0.0001); also, there was a significant difference between the experimental and control groups before and after the study (*P* = 0.0001) (Table 2).

## 4. Discussion

The results indicated that the SQ of participants in the experimental and control groups were greater than 5 before the study. According to the interpretation of the PSQI, the present researchers obtained a total score higher than 5 in the whole, indicating worse sleep quality. Therefore, both groups had a poor SQ and no significant difference was found between them at the beginning of the study, indicating that both groups were homogeneous at the beginning of the study. Consistent with results of the present study, Banack et al. wrote that 52% of participants reported poor SQ in the CR, and it was important to evaluate sleep in CR programs [22]. Lin et al. reported patients with mechanical heart valves suggesting that all patients suffered from poor SQ after a month of admission [16]. Kurose et al. reported the patients' SQ scores higher than 5 in patients with cardiovascular diseases in phase 3 of the CR [23]. Sepahvand et al. examined patients with acute coronary syndrome hospitalized in the intensive care unit and reported that 81% of patients had some degree of SD. Indeed, 50.9% of them had a poor SD, 39.5% had a moderate SD, and 0.5% of samples had a severe SD [15]. Ranjbaran et al. found that the patients' knowledge of causes and methods to overcome poor SQ was low after bypass coronary artery surgery [24]. Based on the results of the present study and other studies, it seems that the SQ of patients after cardiac episodes is poor. Therefore, a comprehensive and complete examination of patients and the development of a care program are necessary to improve the sleep status in cardiac patients.

In the posttest, the overall scores of SQ significantly improved in the experimental group and were less than 5, indicating the good SQ in the patients. In the control group, the quality of sleep improved after the intervention, but the improvement rate was less than the control group and a significant difference was found. Therefore, CR with a special sleep intervention program has been associated with improved SQ in participants. Based on a review of the literature, a lack of information in the intervention was displayed. Lin et al. wrote that the quality of sleep was better in patients using nonpharmacological methods after mechanical heart valve placement than in the control group 5 days after surgery [16]. Ranjbaran et al. found that the SQ was significantly improved after performing the CR program compared to before performing the program (*P* < 0.001) [24]. Martin et al. indicated that a four-session sleep intervention program had beneficial effects on sleep in the elderly [14]. In a study on the effect of rehabilitation on SQ after ablation for atrial fibrillation, Risom et al. wrote that a large number of patients reported low SQ, and the rehabilitation program had no effect on SQ between groups [25]. The results were different due to the different nature of the disease. On the other hand, our special sleep intervention program was also designed and implemented during rehabilitation.

The findings also indicated that SQ was improved and significant differences were found between both experimental and control groups in the pretest and posttest. Despite the higher improvement and no poor SQ in the experimental group, the control group was still weak despite the improvement in SQ. Risom et al. wrote that both groups had improvement in the SQ after rehabilitation in ablation for atrial fibrillation patients [25]. Therefore, it seems that SQ improves over time after hospital discharge after treatment interventions in cardiovascular patients who did not participate in the rehabilitation program.

Our study had several limitations. The first limitation of the study was the method of selecting the control group. These patients did not participate in or dropped out of CR. However, it should be noted that there were many confounding factors such as the lifestyle components influencing the SQ. The second limitation was that PSQI is a subjective measure of sleep, and only a monitored sleep can give objective truth to SQ. Also, the outcome of the study was measured by one scale. The third limitation was the differences in assessment methods (face-to-face versus phone) in the present study that leads to bias in the postscore. Another limitation of this study was treatment fidelity or the uncertainty of the full implementation of interventions taught to patients during sleep. Due to the fact that some patients are not able to participate in the CR program, future studies are suggested investigating the effect of virtual education of sleep improvement interventions on the quality of sleep of patients in need of CR.

## 5. Conclusion

The findings of the present study indicated an improvement in SQ using a sleep intervention program during the CR. However, SQ also improved over time in patients who did not participate in CR, yet this did not lead to acceptable SQ. Therefore, improving patients' SQ requires a team or multidisciplinary performance. Health care providers are advised to provide other care for heart patients, measure their SQ, and make appropriate interventions to improve it. On the other hand, they should use sleep improvement programs as easy and applicable techniques and educate patients. They are also advised to teach health care workers the importance of sleep and its quality and ways to improve it in training programs as essential requirements for human health, especially patients. Further attention should be paid to the CR issue, and rehabilitation centers should be developed to provide facilities for these programs.

## Figures and Tables

**Table 1:** Demographic characteristics of the participants.

Group variable		Experimental group		Control group		*P* value^∗^
		*N*	%	*n*	%	
Sex	Male	23	65.7	25	71.4	0.6
	Female	12	34.4	10	28.6	
Marital status	Single	0	0	34	97.1	0.31
	Married	35	100.0	1	2.9	
Education level	Under high school diploma	27	77.2	25	71.4	0.82
	High school diploma	2	5.7	3	8.6	
	Bachelor and higher	6	17.1	7	20	
Job	Employed	24	6.68	24	6.68	1.000
	Unemployed	11	31.4	11	31.4	
Smoking	Yes	4	11.4	4	11.4	1.000
	No	31	88.6	31	88.6	
Indication for CR	A heart attack	8	22.9	9	25.7	0.85
	A heart surgery	15	42.9	14	40.0	
	A stable heart failure	6	17.1	4	11.4	

^∗^Chi-square.

**Table 2:** Comparison of mean scores of SQ of participants in the experimental and control groups.

Sleep quality	Number	Preintervention		Postintervention		*P* value^∗^
		Mean	SD	Mean	SD	
Experimental group	35	9.2	1.58	4.40	1.14	0.0001
Control group	35	9.02	2.56	7.48	1.86	0.0001
*P* value^∗∗^		00.73		0.0001		

^∗^Paired *t*-test. ^∗∗^Independent *t*-test.

## Data Availability

The datasets used and/or analysed during the current study are available from the corresponding author on reasonable request.

## Abbreviations

SD: Sleep disorders
SQ: Sleep quality
PSQI: Pittsburgh Sleep Quality Index
CR: Cardiac rehabilitation.
